# Efficacy of hybrid remote neuropsychological rehabilitation on cognitive complaints in post-therapeutic lower-grade glioma: the FREEDOME randomized study protocol

**DOI:** 10.3389/fpsyg.2025.1650861

**Published:** 2025-09-23

**Authors:** Estelle Guerdoux, Louise Coutant, Sophie Gourgou, Caroline Mollevi, Marie-Sophie Duc, Fanny Salasc, Hugues Duffau, Amélie Darlix

**Affiliations:** ^1^Department of Palliative and Supportive Care, Montpellier Cancer Institute (ICM), University of Montpellier, Montpellier, France; ^2^Laboratory Epsylon, EA4556, University Paul Valéry, Montpellier, France; ^3^Biometrics Unit, Montpellier Cancer Institute (ICM), University of Montpellier, Montpellier, France; ^4^Institute Desbrest of Epidemiology and Public Health, University of Montpellier, INSERM, CHU Montpellier, Montpellier, France; ^5^Department of Clinical Research and Innovation, Montpellier Cancer Institute (ICM), University of Montpellier, Montpellier, France; ^6^Department of Neurosurgery, University of Montpellier, CHU Montpellier, Montpellier, France; ^7^Department of Medical Oncology, Montpellier Cancer Institute (ICM), University of Montpellier, Montpellier, France; ^8^Institute of Functional Genomics (IGF), University of Montpellier, CNRS, INSERM, Montpellier, France

**Keywords:** lower-grade glioma, neuropsychological rehabilitation and remediation, cognitive behavioral therapy, cognitive complaints, remote telehealth, health-related quality of life, emotional disorders

## Abstract

Lower-grade gliomas (LrGGs) are rare tumors that mainly concern young adults, with an overall survival ranging from 5 to 20 years and without any curative treatment to date. LrGGs are frequently associated with psychological disorders and neurocognitive impairments, including memory and dysexecutive syndrome, that can negatively affect professional, familial and social functioning, ultimately reducing the patients' health-related quality of life (HRQoL). Maintaining an acceptable HRQoL is a key objective because survival outcomes tend to be better in patients with fewer psychopathological comorbidities and better HRQoL. Neuropsychological rehabilitation and cognitive behavioral therapy (CBT) have shown promising results in patients with neurological conditions, mental health disorders, and cancer. However, they are not routinely used in patients with LrGG, and onco-neuro-psychologists (i.e., neuro-psychologists trained in oncology) with expertise in neurocognition and CBT are lacking. The aim of this study protocol is to evaluate the efficacy of the remote FREEDOME program, which integrates CBT and a digital neurocognitive tool, in patients with LrGG who experience cognitive complaints post-therapeutic phase. This ongoing, prospective, open-label, multicenter, randomized trial (1:2) should include 187 patients with LrGG considered “stable” (12 months after surgery, 6 months after chemotherapy or radiotherapy) and who are not planned to receive any cancer treatment in the first 4 months after enrollment. The primary objective is to assess the FREEDOME program effects on perceived cognitive impairment at month 4 post-inclusion. Secondary objectives include patient adherence to the intervention, and effects on the subjective and objective neurocognitive functioning, HRQoL (including sleep, fatigue and return-to-work) and psychopathological symptoms over time. The study will also explore the intervention effects on the patient memory and irritability, as perceived by family caregivers. This study may represent an important step in the implementation of evidence-based neuropsychological interventions tailored to the specific needs of patients with LrGG.

**Clinical trial registration:**

ClinicalTrials.gov: NCT06468176; ID-RCB: 2023-A02376-39.

## 1 Introduction

Although rare, grade 2 and 3 diffuse gliomas, also called lower-grade gliomas (LrGGs) in the 2021 World Health Organization (WHO) classification (Louis et al., 2021), are the most common malignant brain tumor (Low et al., 2022). They are characterized by continuous tumor growth and a median age at diagnosis of 38 years for grade 2, and ~50 years for grade 3 (49 years for anaplastic oligodendroglioma and 52 years for anaplastic astrocytoma) (Ostrom et al., 2023). Overall survival ranges from 5 to 20 years, depending on different factors including histomolecular subtype, tumor volume at diagnosis, resection extent, and tumor growth rate (Ng et al., 2024). Currently, LrGG management remains palliative even for grade 2 tumors that are associated with better overall survival but will ultimately progress (Ng et al., 2024). In this context, one of the main goals is to ensure an acceptable health-related quality of life (HRQoL).

Due to the invasive nature of the disease and the treatment burden, LrGG is often associated with physical problems (e.g., epilepsy) and mental disorders. Psychological distress may gradually decrease over time; however, it remains a frequent complaint. It concerns ~56% of patients before surgery and persists in up to 40% of patients at month 6 after surgery (Rooney et al., 2011; Singer et al., 2018). Long-term studies suggest that such symptoms may continue well beyond the acute treatment phase. For instance, Boele and colleagues reported that 26 years after diagnosis, 23% of patients with LrGG still experienced depression and 53.3% persistent fatigue (Boele et al., 2023). Importantly, adherence to treatment and HRQoL are higher in patients with fewer psychological problems and this may improve their survival outcomes (Mainio et al., 2006). Among the available treatments, cognitive behavioral therapy (CBT) is an evidence-based non-pharmacological intervention (Barlow et al., 2017, 2020; Cuijpers et al., 2025). Few studies have investigated CBT effect on distress and HRQoL in patients with glioma. They demonstrated that comprehensive care based on the CBT principles can alleviate negative psychological states and improve HRQoL (Zhao and Xu, 2021; Boele and Klein, 2018; Liu et al., 2025). Therefore, CBT principles should be integrated in the management of patients with LrGG.

Neurocognitive impairment also is common in patients with LrGG (van Kessel et al., 2017; Schei et al., 2022; Reuvers et al., 2025). It can be observed already at diagnosis, and may, in some cases, lead to the incidental discovery of such tumors (Cochereau et al., 2016). After surgery, clinicians and patients regularly report impairment in one or more cognitive domains (Duffau, 2021). A study found that neurocognitive functioning was stable or improved in 82.8% of long-term survivors with LrGG at 7, 13 and 26 years after diagnosis (Boele et al., 2023). Neurocognitive disorders can significantly disrupt various aspects of the patients' lives, including schooling, employment, return-to-work, family life, and autonomy (Reuvers et al., 2025; Ng et al., 2020; Duffau, 2025; Duffau et al., 2025). In a recent study on 25 patients with grade 2 glioma planned for first-line chemotherapy after their last neurosurgery (median interval = 36 months) (Darlix et al., 2024), 40–53% reported cognitive complaints, using the European Organization for Research and Treatment of Cancer Core 30 (EORTC QLQ-C30) Cognitive Functioning subscale (Aaronson et al., 1993) and the Perceived Cognitive Impairment (PCI) score of the Functional Assessment of Cancer Therapy-Cognitive (FACT-Cog) questionnaire (Lange et al., 2016).

Neurocognitive rehabilitation is effective in several neurological conditions and in different primary cancers. (Bray et al. 2017) assessed a web-based cognitive rehabilitation program (*Insight*) in patients with cancer (excluding brain tumors) and self-reported cognitive symptoms. They found that the FACT-Cog PCI score was significantly improved in the intervention group (compared with the standard care group) at the end of the 15-week intervention, as well as HRQoL outcomes after 6 months. Few already finished or ongoing (NCT03948490) randomized trials focused on LrGG. The randomized trial by (Gehring et al. 2009) showed the promising effect of a face-to-face neuropsychological rehabilitation program on subjective cognitive complaints and objective neurocognitive deficits in 146 patients with “clinically stable” grade 2 glioma (i.e., without any evidence of disease progression for a minimum of 6 months before study entry). However, the implementation of this promising approach is limited by the lack of onco-neuro-psychologists (i.e., neuro-psychologists trained in oncology) (Manso-Ortega et al., 2022). To address this issue, some studies explored the use of digital neurocognitive telerehabilitation programs (van der Linden et al., 2018). Importantly, digital interventions on their own must be proposed at the right time (e.g., relative to treatments, symptoms), and cannot fully replace face-to-face rehabilitation led by a clinical neuropsychologist (van der Linden et al., 2021). Lastly, the other few clinical trials on neuropsychological rehabilitation in LrGGs have several limitations, such as small sample size (van der Linden et al., 2021; Locke et al., 2008; Richard et al., 2019; Yang et al., 2014; Maschio et al., 2015), heterogeneous populations (van der Linden et al., 2021; Yang et al., 2014; Zucchella et al., 2013), and/or methodological weakness (Maschio et al., 2015; Hassler et al., 2010; Miotto et al., 2014).

The aim of this French multicenter randomized study is to evaluate the impact of a hybrid neuropsychological rehabilitation program, which combines CBT delivered by a trained onco-neuro-psychologist with a digital neurocognitive training tool, in patients with LrGG and cognitive complaints during the post-therapy period.

## 2 Methods and analysis

### 2.1 Study design and settings

**Figure 1:**
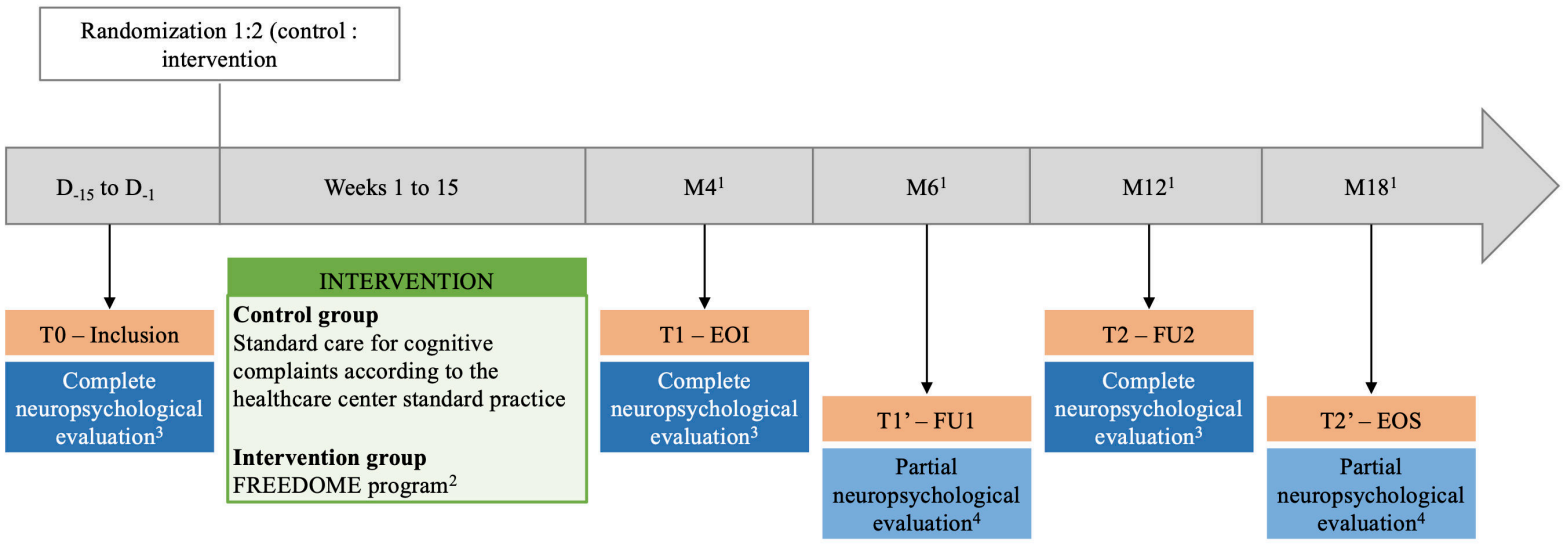
The FREEDOME study timeline. ^1^Follow-up periods: month (M) 4, 6, 12, and 18 after inclusion (±2 weeks). ^2^Cognitive behavioral therapy by telehealth sessions (at least three sessions, maximum one session/week) and BrainHQ digital program (4 × 40-min session/week). ^3^Assessment of cognitive complaints, psychopathological functioning, patient's quality of life, fatigue, and neurocognitive functioning. ^4^Assessment of cognitive complaints, psychopathological functioning, patient's quality of life, and fatigue. D, day; FU, follow-up, EOI, end of intervention; EOS, end of study.

**Figure 2:**
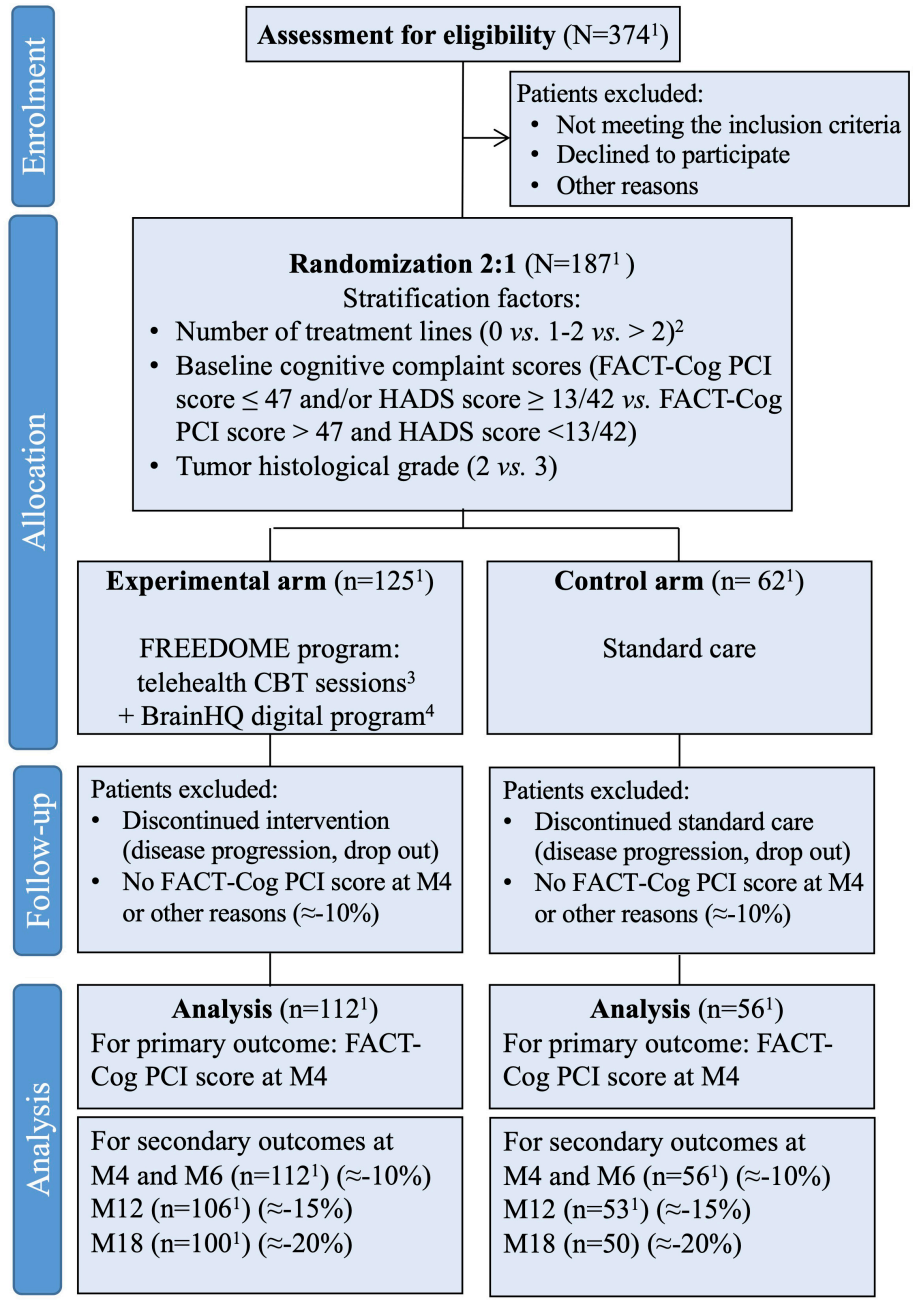
Flow diagram of the study FREEDOME. ^1^Expected number of patients. ^2^Chemotherapy or radiotherapy. ^3^Performed by a trained onco-neuro-psychologist (max 1 session/week for 15 weeks). ^4^Four-month program (Insight from Posit Science BrainHQ, San Francisco, California, 357 USA). FACT-Cog, Functional Assessment of Cancer Therapy-Cognitive Scale; PCI, Perceived Cognitive Impairment sub-score; HADS, Hospital Anxiety and Depression Scale.

The FREEDOME study (*E*ff*icacité d'un programme mixte de* ré*habilitation n*e*uropsychologique en* d*istanciel chez* d*es patients porteurs d'un gli*ome *diffus de grade 2 ou 3: essai randomisé contrôlé*, Efficacy of a hybrid neuropsychological telerehabilitation intervention on cognitive complaints in patients with LrGG after therapy) (NCT06468176) is an ongoing, prospective, open-label, multicenter, randomized controlled trial carried out in France. Patient recruitment is open since June 2024. The study is expected to last 42 months in total: 24-month enrollment period and 18-month follow-up phase. Patient will be included through a two-step process that includes screening and inclusion visits. Eligible patients who consent to participate will sign a written informed consent (Supplementary material) before enrollment. Patients will be randomly allocated to the control arm (standard care) or the intervention arm (FREEDOME program) (1:2 ratio). Figure 1 shows the study timeline and Figure 2 the study flowchart. Table 1 presents the data collection schedule. This study follows the Standard Protocol Items: Recommendations for Interventional Trials (SPIRIT) checklist to ensure the quality of the study protocol.

**Table 1:** Detailed data collection schedule.

**Data collected**	**Screening inclusion**	**Intervention**	**EOI visit (T1)**	**FU visit (T1^′^)**	**FU visit (T2)**	**EOS visit (T2^′^)**
	**D**_−15_ **to D**_−1_	**W1–15**	**M4** ±**2W**	**M6** ±**2W**	**M12** ±**2W**	**M18** ±**2W**
Eligibility criteria	✓					
Consent form signature	✓					
Randomization	✓					
Cognitive complaints (patient- ± caregiver-rated FACT-Cog, PRMQ)	✓		✓	✓	✓	✓
Psychological functioning (patient- ± caregiver-rated HADS, BDI-II, STAI-Y, BITe)	✓		✓	✓	✓	✓
Quality of life (EORTC QLQ-C30, BN20)	✓		✓	✓	✓	✓
Fatigue (MFI-20, ISI)	✓		✓	✓	✓	✓
Neurocognitive functioning (MoCA, WAIS-IV subtests, HVLT, SENTB, RMET)	✓		✓		✓	
CBT by videoconference (at least 3 sessions, maximum 1 session/week)		✓^a^				
BrainHQ program (4 × 40-min session/week)		✓^a^				

BDI-II, Beck Depression Inventory-II; BITe, Brief Irritability Test; CBT, Cognitive Behavioral Therapy; EORTC QLQ-C30 and BN20, European Organization for Research and Treatment of Cancer Core 30 and Brain-20; FACT-Cog, Functional Assessment of Cancer Therapy-Cognitive questionnaire; HADS, Hospital Anxiety and Depression Scale; HVLT, Hopkins Verbal Learning Test; ISI, Insomnia Severity Index; MoCA, Montreal Cognitive Assessment; PRMQ, Prospective and Retrospective Memory Questionnaire; RMET, Reading the Mind in the Eyes Test; SENTB, Standard Executive Neuropsychological Test Battery; STAI-Y, State-Trait Anxiety Inventory-Y; WAIS-IV, Wechsler Adult Intelligence Scale, Fourth Edition, subtests Coding and Symbol Search.

D, day; W, week; FU, follow-up, EOI, end of intervention, EOS, end of study.

^a^Experimental group only.

### 2.2 Study objectives

The primary objective is to assess the impact of the FREEDOME program on the perceived cognitive impairment in patients with LrGG at month 4 (M4) after the intervention. Secondary objectives include assessing the patient adherence, and evaluating the intervention effect on subjective cognitive and memory complaints, HRQoL (particularly, sleep, fatigue, return-to-work) and psychological problems at M4, M6, M12 and M18 after the intervention. The study will also assess neurocognitive functioning at baseline, M4 and M12, and investigate the relationship between subjective and objective cognitive outcomes. Lastly, exploratory objectives will focus on evaluating the longitudinal impact of the intervention on memory and irritability as perceived by family caregivers at M4, M6, M12, and M18.

### 2.3 Endpoint measures

The primary endpoint is the perceived cognitive impairment, assessed using the FACT-Cog PCI score (Lange et al., 2016) at M4. The self-report FACT-Cog questionnaire is used to assess subjective cognitive complaints and has been validated in patients with cancer. It also evaluates the cognitive impairment effect on HRQoL. It has a good internal consistency reliability (Cronbach's alpha between 0.74 and 0.91) and good convergent validity (significant correlations with the scores of scales assessing anxiety and depression).

Secondary endpoint measures will include:

- Patient adherence (i.e., completing at least 25 h of the recommended 40 h of the digital program and attending three telehealth consultations). The 25-h threshold is based on the study by (Bray et al. 2017), where the FACT-Cog PCI score change was higher in participants who trained >25 h than in those who trained <25 h (16.3 vs. 12.6 points). The three telehealth sessions (at the intervention start, mid-point, and end) will ensure sufficient therapeutic support, address barriers, and will allow reviewing progress and delivering personalized recommendations.
- Comparison of the FACT-Cog PCI scores (Lange et al., 2016) at baseline, M6, M12, and M18.
- Comparison of the scores the three other FACT-Cog subscales at baseline, M4, M6, M12, and M18.
- Comparison of the Prospective and Retrospective Memory Questionnaire (PRMQ) scores (Guerdoux-Ninot et al., 2019) at baseline, M4, M6, M12, and M18.
- Comparison of the EORTC QLQ-C30 scores (Aaronson et al., 1993) and its module Brain-20 (BN20) scores (Taphoorn et al., 2010) at baseline, M4, M6, M12, and M18.
- Comparison of the 20-item Multidimensional Fatigue Inventory (MFI-20) (Gentile et al., 2003) and the Insomnia Severity Index (ISI) (Savard et al., 2005) scores at baseline and M4, M6, M12, and M18.
- Proportion of patients returning to work or increasing their work hours at M4, M6, M12, and M18.
- Changes in self-rated psychological distress, depression, anxiety and irritability between baseline and M4, M6, M12, and M18 using the Hospital Anxiety and Depression Scale (HADS) (Razavi et al., 1990), Beck Depression Inventory-II (BDI-II) (Beck et al., 1996), State-Trait Anxiety Inventory-Y (STAI-Y) (Bellaert et al., 2022), and Brief Irritability Test (BITe) (Bellaert et al., 2022), respectively.
- Differences (between baseline and M4 and M12) in the clinician-rated scores of the following neurocognitive tests: Montreal Cognitive Assessment (MoCA) (Nasreddine and Patel, 2016), Working Memory Index [score derived from the Digit Span and Arithmetic subtests of the Wechsler Adult Intelligence Scale, Fourth Edition (WAIS-IV)], Processing Speed Index (from the Coding and Symbol Search subtests of the WAIS-IV) (Wechsler, 2011), Hopkins Verbal Learning Test (global scores) (Rieu et al., 2006), Standard Executive Neuropsychological Test Battery (Bayard et al., 2023) (including the modified Stroop Delis-Kaplan test, the Digit Span subtest of the WAIS-IV, the Verbal Fluency Test, the Trail Making Tests A and B), and Reading the Mind in the Eyes Test (Prevost et al., 2014).
- Correlations between the FACT-Cog sub-scores or PRMQ scores and key neurocognitive domains (global functioning, executive functions, language, working memory, processing speed, and social cognition) at baseline, M4, and M12.

Exploratory secondary endpoints will include:

- Family caregiver-rated PRMQ scores at M4, M6, M12, and M18 (Guerdoux-Ninot et al., 2019).
- Family caregiver-rated BITe scores at baseline and M4, M6, M12, and M18 (Bellaert et al., 2022).

### 2.4 Eligibility criteria

#### 2.4.1 Inclusion criteria

- Age ≥ 18 years.
- Histomolecular diagnosis of LrGG according to the 2021 WHO classification, regardless of prior oncological treatments.
- Eastern cooperative oncology group performance status (ECOG-PS) score ≤ 2.
- Neurosurgical resection (excluding biopsy) performed ≥12 months before inclusion.
- Cancer treatment (chemotherapy or radiation therapy) completed at least 6 months before inclusion.
- Cognitive complaints defined at least as “*Quite*” on items 20 and/or 25 of the EORTC QLQ-C30 questionnaire (i.e., “Cognitive Functioning” subscale score ≤ 66.67).
- Patient fluent in French.
- Patient covered by the French Social Security system.
- Regular use of digital tools and internet access.
- Signed informed consent.

#### 2.4.2 Non-inclusion criteria

- Vision or hearing impairment preventing the use of computer tools (lateral homonymous hemianopia is not an exclusion criterion).
- Participation in another study with neurocognition as the primary outcome.
- Legal incapacity or conditions preventing consent obtention or study completion.
- Unstable psychiatric disorder (psychotropic drugs are not an exclusion criterion if doses are stable).
- Severe cognitive impairment (e.g., neurodegenerative disease, sequelae of traumatic brain injury), MoCA score ≤ 20/30 (Nasreddine and Patel, 2016), or impaired ability to use digital tools at home.
- Planned oncological treatment within 4 months of inclusion (isocitrate dehydrogenase- targeted therapies are allowed).

### 2.5 Sample size

To identify clinically meaningful improvements, responders will be defined as patients who show a FACT-Cog PCI score reduction of at least 6.5 points between baseline and M4. This threshold is based on previously validated minimal clinically important differences observed in patients with cancer, and estimated from the results of the TémoIN trial (NCT03257618) (Darlix et al., 2024) and the study by (Bray et al. 2017). A sample size of 168 evaluable patients is required to detect a 6.5-point mean difference in the FACT-Cog PCI score (control: 50, intervention: 56.5) with 80% power, two-sided α = 0.05, and an assumed standard deviation of 14 (Darlix et al., 2024; Bray et al., 2017). To take into account drop-outs, a conservative attrition rate of 10% was considered for this clinically stable LrGG population. This rate is consistent with attrition benchmarks in glioma trials, but it is likely to overestimate losses in cohorts of patients with clinically stable LrGG that are generally characterized by higher retention. Participation burden (many questionnaires at multiple time points) was also taken into account.

Therefore, 187 patients will be enrolled (187 × 0.90 = 168 patients evaluable at M4 for the primary endpoint). Based on prior observations from the TémoIN study (NCT03257618) showing that ~50% of screened patients were eligible, 374 patients will be screened over 24 months to reach the planned sample size (*n* = 187).

For the secondary endpoints, cumulative attrition is conservatively projected as follows: =10% by M6, =15% by M12, and =20% by M18. These estimates reflect modest progressive losses to follow-up, but also take into account the relative functional stability of patients with LrGG and the planned retention strategies (flexible schedule, family caregiver engagement, proactive follow-up). Sample size targets and the resulting evaluable number of patients were adjusted to align with these timepoint-specific attrition expectations, ensuring sufficient power for the primary and secondary analyses. Using these estimates, the expected number of evaluable participants at each scheduled assessment is reported in Figure 2.

### 2.6 Randomization

Patients will be randomized in a 2:1 ratio (intervention: control) using a random block randomization method. This approach will maximize the number of patients who can benefit from the FREEDOME program. Stratification factors include:

- Number of treatment lines (chemotherapy or radiotherapy, 0 vs. 1–2 vs. > 2);
- Baseline FACT-Cog PCI score (cognitive complaints) and HADS (distress) score (PCI score ≤ 47 and/or HADS score ≥13/42 vs. PCI score >47 and HADS score <13/42);
- Tumor histological grade (grade 2 vs. 3).

Investigators will register patients using the online electronic case report form (Ennov Clinical^®^).

### 2.7 Intervention protocol

At the screening visit, the investigator (a neuro-oncologist or an onco-neuro-psychologist) will confirm the patient's eligibility criteria and collect the informed consent form.

At the inclusion telehealth session, the onco-neuro-psychologist will collect demographic data, symptoms, rehabilitation status, concomitant treatments, disease history, prior oncological treatments, tumor laterality and eloquence, seizure frequency, time since last treatment, and scores from self-rated questionnaires and from clinician-rated neurocognitive tests. A comprehensive neuropsychological assessment will be performed, focusing on cognitive complaints, psychopathological states, HRQoL, fatigue, and objective neurocognitive functioning. The patient will then be randomized.

#### 2.7.1 Intervention period

In the experimental arm, patients will follow the FREEDOME program that combines CBT with a onco-neuro-psychologist and the 4-month BrainHQ digital program, as detailed in the “FREEDOME program” section. In the control group, cognitive complaints will be managed according to the healthcare center's standard practice. This may include routine follow-up, psychological support, or speech therapy, depending on resource availability and the investigator's recommendations. Digital cognitive rehabilitation will not be available to these patients until study completion.

#### 2.7.2 Follow-up period

The onco-neuro-psychologist will conduct an end-of-intervention telehealth visit at M4 (±2 weeks) post-inclusion. This visit will include a comprehensive neuropsychological assessment covering cognitive complaints, psychological problems, HRQoL, fatigue, sleep, work activity changes, and neurocognitive functioning. Feasibility is supported by our previous longitudinal study in a similar population (Darlix et al., 2024), in which patients successfully completed lengthy neuropsychological assessments (~3 h). Two additional follow-up telehealth visits will be carried out at:

- M6 (T1′): Assessment of cognitive complaints, psychopathological functioning, HRQoL, fatigue, and sleep;
- M12 (T2): Comprehensive neuropsychological assessment (like at the inclusion and end-of-intervention visits).

The study will end at M18 post-inclusion after the T2′ telehealth visit during which the onco-neuro-psychologist will carry out a neuropsychological evaluation focusing on cognitive complaints, psychopathological functioning, HRQoL, fatigue and sleep. Figure 1 outlines the study timeline, and Table 1 presents the data collection schedule.

### 2.8 FREEDOME program

#### 2.8.1 Cognitive behavioral therapy

CBT sessions will be conducted remotely (telehealth sessions) by the same trained onco-neuro-psychologist with expertise in CBT at all centers to ensure consistency. Each patient will be offered at least three CBT sessions (maximum one per week over 15 weeks) to address cognitive psychopathology, emotional regulation, fatigue and sleep management, and give support for the digital cognitive rehabilitation program. The aim is to improve the patients' perception and interpretation of situations, fostering adaptive cognitive, emotional, and behavioral responses. During these sessions, the onco-neuro-psychologist will propose to assess cognitive distortions and individual cognitive profiles, followed by the development of strategies to increase emotional awareness, improve tolerance, enhance control, and foster adaptive coping behaviors.

Besides the digital program support, the telehealth sessions will focus on key determinants of fatigue and sleep. Fatigue and insomnia are very frequently reported by patients with cancer, and could have important psychosocial and emotional consequences, such as reducing daily activities and HRQoL (Baussard et al., 2022; Morin et al., 2006). In these sessions, factors such as cancer-related adjustments, fear of tumor progression, dysfunctional fatigue-related cognition, and negative social interactions will be discussed. If needed, the intervention will incorporate insomnia treatment components, such as sleep hygiene and sleep restriction, as developed in on-line insomnia-CBT interventions (Boinon et al., 2021, 2024).

Psychopathology exists on a continuum with normal emotional reactions, but is characterized by exaggerated or inappropriate responses. The cognitive model of psychopathology suggests that information processing distortions and emotional intolerance are similar in patients with brain tumors and in patients with depression, anxiety disorders, and other psychiatric conditions (Barlow et al., 2017, 2020; Farchione et al., 2012; García-Torres et al., 2022). Additionally, patients with brain tumors may exhibit vulnerabilities and idiosyncratic sensitivities that reflect their cognitive structures, predisposing them to psychological distress linked to perceived threats (Trouillet et al., 2019). In this study, the onco-neuro-psychologist will use CBT to help patients to develop adaptive responses that will allow them to perceive reality accurately and respond with appropriate behaviors. Throughout the course of treatment, the domains of thoughts, physical sensations, and behaviors will be examined in depth, with a specific focus on identifying maladaptive emotion regulation strategies within each of these domains. The objective of the intervention is to replace these strategies with more adaptive emotion regulation skills.

Depending on the emotional symptom level reported by the patient, five key areas will be addressed during the consultations, following the Unified Protocol For Transdiagnostic Treatment Of Emotional Disorders proposed by (Barlow et al. 2020) to ensure standardization and preserve clinical flexibility. This protocol has been widely validated and is adaptable to medical settings. This framework provides a structured sequence of core modules (psychoeducation, cognitive reappraisal, exposure, relapse prevention). We added complementary modules (CBT for insomnia and fatigue management), when clinically indicated.

In summary, the intervention is based on CBT components with demonstrated effectiveness that specifically target negative emotionality and maladaptive responses to emotions when they occur. It begins with an introductory session to review the patient's symptoms and provide a therapeutic rationale. This is followed by a module focused on motivational enhancement and another dedicated to psychoeducation about emotions. The intervention concludes with a relapse prevention module.

This approach may allow us to address both transdiagnostic emotional symptoms and specific issues encountered by patients with LrGG. Sessions will be standardized at all centers, with a minimum of three consultations and flexibility for additional sessions (up to one per week) for 4 months, depending on the patient's clinical profile. Tables 2, 3 present the CBT syllabus (overview and long version, respectively) for transparency and reproducibility.

**Table 2:** CBT syllabus (overview).

**Session(s)**	**Core content**	**Theoretical framework**	**Clinical objectives**
Introductory session (mandatory)	Symptom review, therapeutic rationale, motivational enhancement, functional analysis (cognition, fatigue, sleep, emotional distress)	Unified Protocol (Barlow), CBT model of psychopathology	Build therapeutic alliance, clarify treatment goals, enhance motivation
Core sessions (≥2 mandatory; additional weekly sessions for up to 15 weeks, if clinically indicated)	Psychoeducation on emotions; assessment of cognitive distortions; cognitive restructuring; behavioral activation; fatigue- and sleep-focused strategies (CBT-I components: sleep hygiene, stimulus control, sleep restriction if needed); adaptive coping skills training	Unified Protocol (psychoeducation, cognitive restructuring, emotion regulation); CBT-I (as indicated)	Increase emotional awareness, challenge maladaptive cognitions, improve sleep/fatigue management, foster adaptive coping
Final/closure session (mandatory)	Relapse prevention, consolidation of the learned skills, recommendations for daily life and digital tool use	Unified Protocol (relapse prevention), CBT-I (maintenance)	Consolidate adaptive responses, enhance autonomy, reduce relapse risk

**Table 3:** CBT syllabus (details).

**Module**	**Main objectives**	**Content/Techniques**	**Adaptations to LrGG**
1. Introduction and motivation	Assess symptoms and expectations; build alliance; enhance motivation	Motivational interviewing; goal setting; program overview	Link to cancer history and treatment experience
2. Psychoeducation on emotions	Understand adaptive role of emotions; differentiate normal vs. pathological responses	Psychoeducation; thought–emotion–behavior cycle	Applied examples: fear of progression, fatigue, irritability
3. Emotional awareness	Increase attention to emotional experience; reduce avoidance	Emotion observation; emotion journal	Adapted to cognitive limitations or impairments (guidance, visual aids)
4. Cognitive reappraisal	Identify cognitive distortions; reappraise automatic thoughts	Dysfunctional thought detection; reappraisal exercises	Target fatigue-related cognitions, memory beliefs, fear of relapse
5. Avoidance and emotion-driven behaviors	Identify avoidance; reduce maladaptive behaviors	Functional analysis; alternative behaviors	Examples: social withdrawal, avoidance of stimulating activities
6. Graduated exposure	Increase tolerance to sensations and anxiety-provoking situations	Interoceptive and situational exposures	Examples: return to work, fatigue, memory difficulties
7. Complementary modules	Address fatigue and insomnia	CBT-I: sleep hygiene, sleep restriction, relaxation; activity pacing	Added if major sleep complaints
8. Relapse prevention	Consolidate skills; prepare for relapse	Relapse prevention plan; progress review; self-prescription	Focus on autonomy and long-term maintenance

#### 2.8.2 BrainHQ digital application

The Brain HQ digital application (Insight from Posit Science Brain HQ, San Francisco, California, USA) is a digital neurocognitive training program designed based on the principles of neuroplasticity. It was developed by an international team of neuroscientists led by Prof. Michael Merzenich with the objective of improving cognitive functions through structured exercises. Participants will train for 60–160 min per week, divided into 1–4 sessions of 40–60 min/each. The recommended training regimen consists of four 40-min sessions per week for 15 weeks (total = 40 h). A detailed overview of the exercises included in the application is provided in Table 4.

**Table 4:** Detailed description of the BrainHQ digital program.

**Exercise**	**Content**
Double decision	This module leverages a unique, proven technology designed to accelerate processing speed and expand the useful field of vision. Commonly referred to as “speed training” in research, this technology has been used in many studies that demonstrate benefits such as faster visual processing, broader field of view, enhanced driving safety, and more.
Follow the target	Participants are required to divide their visual attention and track multiple moving objects simultaneously. The target tracker is designed to encourage patients to divide their focus by challenging them to follow several objects that move at the same time on the screen.
Visual scanning	This module trains patients to identify without effort specific visual patterns at high speeds, enhancing their overall visual acuity. The key skill targeted by this module is visual processing speed. During the exercise, the color, direction, and thickness of the scanning bars will vary to challenge the participants.
Attention to detail	Designed to improve patients' ability to make quick, precise saccades, this module trains the ability to notice subtle details with each eye movement. It focuses on quickly and reliably extracting information from each visual fixation.
Hawk eye	This module enhances visual accuracy, enabling patients to see details rapidly and precisely. This improves memory encoding, allowing patients to better retain the information they encounter (e.g., from a film, a wedding, house hunting, birdwatching). The ability to remember these details accurately helps them to discuss these experiences effectively in the future. The aim of this module is to improve everyday life activities.

### 2.9 Modification or discontinuation of the allocated intervention

Premature withdrawal may occur due to the patient's or investigation's decision, loss to follow-up or death. It may also occur due to disease progression that requires new treatments within 4 months of inclusion, disease progression after the intervention period (M4 to M18 post-inclusion), a new neurological deficit that hinders BrainHQ use (e.g., motor or visual impairment, excluding homonymous hemianopia), a severe emotional event (e.g., death of a close relative or traumatic event requiring psychological care), a new cancer, or major protocol deviations. In this case, the patient is allowed to use the Brain HQ digital application as often as wanted, and to ask support from the onco-neuro-psychologist, until M4.

### 2.10 Concomitant care

Psychotropic medications (antidepressant, anxiolytic, hypnotic, antihistaminic, antipsychotic, anti-epileptic drugs and corticosteroids) will be permitted during the study, if clinically indicated. These treatments, class and dose, will be recorded in the electronic case report form, and analyses will be adjusted for these factors. As patients enrolled in the study are required to be in a stable post-therapy phase, minimal changes in drug dosage or regimen are expected during the study period, regardless of the randomization arm. This reduces the risk of bias due to intercurrent pharmacological modifications.

### 2.11 Safety

In this study, potential adverse events are expected to be mild, transient, and non-serious. Some participants may report temporary visual fatigue or slight headache related to the use of digital tools, including telehealth consultations and/or the digital cognitive rehabilitation program. These events are expected to resolve spontaneously and have no long-term consequence. All adverse events will be reported through the appropriate health vigilance systems in compliance with the current regulations. The investigator will inform the study sponsor (Montpellier Cancer Institute, France) about all new safety data that could influence the study benefit-risk assessment or will require updates to study documents. All patient safety concerns identified during the study period must be reported to the sponsor without delay.

### 2.12 Data collection

Patients will have access to a dedicated digital application (“*My trial Patient*”, Hoppen) to complete self-rated questionnaires (thus excluding clinician-rated neurocognitive assessments). Upon inclusion, they will receive a user guide and/or a trained staff member will assist them in accessing and using the application to ensure independent and accurate questionnaire completion. Patients will receive SMS reminders about the scheduled questionnaire completion dates. Patients who do not like to use digital applications will be able to complete the questionnaires in paper format.

Some assessments (i.e., BDI-II and STAI-Y questionnaires) will be provided in paper format by default with the material for specific neuropsychological tests (i.e., Visuospatial/Executive subtest of the MoCA, Symbol Search and Coding subtests of the WAIS-IV, and Trail Making Tests). Participants will be asked not to open the documents before the consultation time.

Remote testing procedures will be standardized. The onco-neuro-psychologist will administer all neuropsychological tests remotely via a telehealth platform. Each assessment will last between 1 and 1.5 h. Tests will always be administered in the same order, following strict standardized instructions, as specified in the manual of each cognitive test used (see references for each test). For paper-based tests (i.e., the Visuospatial/Executive subtest of the MoCA, Symbol Search and Coding subtests of the WAIS-IV, and Trail Making Tests), patients will complete the exercises at home under direct remote supervision by the onco-neuro-psychologist. Upon completion of each paper-and-pencil test, patients will show their work on screen, and the onco-neuro-psychologist will take a screenshot for record purposes. Patients are also instructed to return the completed tests by post to the study coordinating center using the provided pre-stamped and pre-addressed envelopes. All assessments will be conducted in accordance with the best practices in neuro-psychology and the standardized administration procedures specific for each test.

The patient engagement with the Brain HQ digital application will be monitored through automated usage metrics. Patients will not be constrained or directly supervised in their use; however, the onco-neuro-psychologist can address adherence issues during the telehealth CBT sessions if needed.

If both patient and family caregiver agree, the family caregiver will provide an estimation of the patient's irritability and memory errors in daily life, by filling in the QMRP and BITe questionnaires (paper or digital version) at the same timepoints.

CBT sessions will not be recorded; however, the intervention fidelity and standardization will be ensured through regular supervision by a senior clinician, who will monitor the adherence to the manualized syllabus. All sessions will be delivered by the same trained onco-neuro-psychologist at all centers, ensuring consistency. In the rare event of absence, a senior clinician trained in CBT, neuropsychology and oncology will conduct the session, following the manualized syllabus. These measures provide structured oversight and preserve the intervention fidelity throughout the study.

In addition, baseline characteristics, compliance to the intervention (e.g., intervention adherence, session exposure, digital engagement) and other potential confounding parameters, such as psychotropic and anti-seizure drugs, corticosteroids, tumor laterality and eloquence, seizure frequency, and time since last treatment, will be collected/recorded in the electronic case report form to detect possible confounding effects.

### 2.13 Data analysis

A statistical analysis plan will be drafted before database locking. For the for primary endpoint, all statistical analyses will be done using the intention-to-treat population and the evaluable population (i.e., patients with a FACT-Cog PCI score at baseline and M4).

Frequencies (N) and percentages (%) will be used to describe qualitative variables, and missing data reported. Percentages will be calculated after excluding missing data. Number of observations (N), median, minimum, maximum, mean, standard deviation, and interquartile range will be used to describe quantitative variables.

Baseline demographic and clinical characteristics, interventions and concomitant treatments will be described.

Questionnaire scores will be calculated according to established guidelines: FACT-Cog (Lange et al., 2016), QLQ-C30 and BN20 (EORTC manual), PRMQ (Guerdoux-Ninot et al., 2019), and neurocognitive and psychopathological tests (Gentile et al., 2003; Savard et al., 2005; Razavi et al., 1990; Beck et al., 1996; Bellaert et al., 2022; Nasreddine and Patel, 2016; Wechsler, 2011; Rieu et al., 2006; Bayard et al., 2023; Prevost et al., 2014; Spielberger, 1983; Guilmette et al., 2020). Scores will be reported and missing data rates recorded at all time points. Adherence rates will be estimated with their 95% confidence intervals.

The primary endpoint (FACT-Cog PCI score at M4) will be compared between arms with the Student's *t*-test or Wilcoxon/Mann-Whitney rank-sum test. An ANOVA or ANCOVA model will be used to adjust for potential confounders. A 5% significance threshold will be applied.

For the secondary endpoints:

- the FACT-Cog PCI scores will be compared between arms and time points using a similar method. A 5% significance threshold will be applied with adjustments for multiple comparisons to control the alpha risk using the Benjamini–Hochberg method (Benjamini and Hochberg, 1995). All exploratory analyses will be performed without adjustment.
- Partial correlations between the FACT-Cog or PRMQ subscale score and key cognitive domains will be evaluated using Spearman or Kendall Tau correlation coefficients, adjusted for distress (HADS) and fatigue (MFI-20, ISI). Each neuropsychological threshold will be defined according to the test guideline and taking into account many variables, such as age, sex and/or socio-cultural level.
- Other questionnaire scores will be compared between arms with the Student's *t*-test or Wilcoxon/Mann-Whitney rank-sum test. An ANOVA or ANCOVA model will be used to adjust for potential confounders.

Within each arm, score differences (HADS, PRMQ, BDI-II, STAI-Y, BITe, MFI-20, ISI, QLQ-C30, and BN20) between baseline and the subsequent timepoints will be assessed using the paired Student's *t* or Wilcoxon signed-rank test.

First, the mean score change over time will be graphically represented using the LOcally Estimated Scatterplot Smoothing method (LOESS).

Then, Linear Mixed Models (LMMs) may be used to model the score changes over time in function of the group. These LMMs will include random intercepts and slopes, assuming a linear trajectory over time. If necessary, more flexible LMMs (e.g., spline-based models) may be considered.

Although the open-label design may expose the study to attention and expectancy biases, particularly regarding self-reported outcomes, the data analysts involved in the primary outcome analysis will remain blinded to group allocation.

### 2.14 Data management, quality and monitoring, and responsibilities

The study sponsor is responsible for the study design and management, and ethical compliance. It will secure the necessary authorizations, ensure insurance coverage, notify the authorities about the study milestones, generate the final report, and keep study documents for at least 15 years. It will oversee data quality, analysis, confidentiality, and storage. Participant data will be available upon request, subject to a contract. The study protocol, statistical analysis plan, and analytical code may also be shared under a data transfer agreement following the European Union General Data Protection Regulation. Data monitoring will follow the same sponsor-defined plan at all centers, with informed consent verification as a minimum requirement. The sponsor and relevant authorities may audit the trial for at least 15 years after completion.

## 3 Discussion

LrGGs represent a unique neuro-oncological challenge due to their chronic course, frequent onset in young adulthood, and the associated neurocognitive and psychological burdens. In the absence of curative treatments, optimizing long-term functioning and preserving HRQoL remain essential therapeutic goals. Neurocognitive and emotional problems are common in patients with LrGG, and their impact on the daily life (professional, familial, and social) may be important (Reuvers et al., 2025; Ng et al., 2020; Duffau, 2025; Duffau et al., 2025). Despite the growing recognition of these issues, neuropsychological care is not systematically integrated in LrGG management. The present study protocol addresses this unmet clinical need by evaluating a multimodal intervention (the FREEDOME program) that combines clinician-led CBT and a digital neurocognitive rehabilitation tool, in addition to the usual neuro-oncological care (e.g., seizures management).

Several studies highlighted the strong interconnection between distress, supportive care needs, and overall HRQoL. Lower HRQoL scores reflect greater distress and higher care demands (van Kessel et al., 2017; Snyder et al., 2008). Behavioral and emotional disorders as well as the uncertainty about prognosis should be taken into account and managed by clinicians in order to improve the patients' wellbeing. Yet, survival remains the primary outcome in most clinical trials, including in patients with glioma. Moreover, studies on CBT effect on distress and HRQoL in patients with glioma are rare and have some limitations (Zhao and Xu, 2021; Boele and Klein, 2018; Liu et al., 2025). Samples were often heterogeneous (patients with grade 2, 3 or 4 gliomas). CBT was provided by nurses or Internet-based guided self-help supervised by a coach (a researcher psychologist or a psychology student), instead of being delivered by psychologists specialized in cancer. None of the previous studies investigated the specific effect of CBT on cognitive complaints. Yet, cognitive and emotional function deficits are two of the main contributors to HRQoL alterations in patients with LrGG (Liu et al., 2025). Therefore, the aim of this study protocol is to assess whether CBT, delivered by a psychologist trained in CBT, oncology and neuropsychology (onco-neuro-psychologist), is a relevant key care component in patients with LrGG. Distress management through CBT sessions could alleviate cognitive complaints and improve HRQoL.

In a previous randomized controlled trial in patients with glioma and cognitive complaints and disorders, participation in a face-to-face cognitive rehabilitation intervention significantly improved performance in memory and attention tests and reduced cognitive complaints and mental fatigue (Gehring et al., 2009, 2010). To enhance the accessibility to this rehabilitation program (Gehring et al., 2011) in a cost-effective and user-friendly manner, the authors developed a tablet-based version (*ReMind*). A preliminary pilot study demonstrated the feasibility of delivering cognitive telerehabilitation using *ReMind* to adults with LrGG or meningioma after surgery (van der Linden et al., 2018). However, in the subsequent randomized controlled trial in patients with a brain tumor, differences in mean scores, percentages of patients with cognitive impairment and reliable change indices did not reach the significance between groups (*ReMind* vs. waiting list) (van der Linden et al., 2021). The authors blamed particularly the recruitment difficulties, resulting in limited statistical power. The timing may have played a major role: the telerehabilitation intervention was proposed to patients 3 months after surgery. However, as underlined also by the authors, during this phase, patients require time for physiological recovery (and in some cases, adjuvant therapy) and for adapting to their diagnosis and associated symptoms, and often prioritize reintegration into familial, occupational, and social environments. Thus, this early intervention may have not met the patients' needs. In this trial, telephone assistance was provided by a researcher who addressed the patients' questions, difficulties, and experiences.

We think that the complexity of cognitive and emotional problems experienced by patients with LrGG often exceeds the capacities of purely digital tools or simple telephone contacts. Personalized and tailored support remains crucial for fostering patient adherence, building a strong therapeutic confidence, and addressing psychological-oncological issues (i.e., distress and emotional disorders) that in turn, influence both objective and subjective neurocognitive functioning (Darlix and Guerdoux, 2024; Nicol et al., 2019; Noll et al., 2017). Moreover, we think that neuropsychological rehabilitation should be offered during periods of psychiatric and medical “stability”, and after recovery from intensive treatments (Weyer-Jamora et al., 2021). Its aim should be to reduce the gap between the patient/environment/responsibility expectations and the patient abilities/skills, while changing the perception of both.

For these reasons, we propose an innovative and hybrid neuropsychological telerehabilitation intervention that combines digital support and individualized interventions delivered by a onco-neuro-psychologist and that should be carried out in a post-therapeutic “stable “period (i.e., >12 months after surgery or >6 months after chemotherapy or radiotherapy, without any oncological treatment planned in the next 4 months). This should enhance the ecological validity and user engagement, but also preserve the therapeutic alliance and clinical expertise needed to identify and address emotion regulation difficulties and cognitive dysfunctions.

A key strength of this study is that all CBT sessions will be conducted by the same trained onco-neuro-psychologist at all centers to ensure consistency (with regular supervision by a senior clinician to maintain fidelity to the manualized protocol). This approach also addresses the scarcity of psychologists trained in neuro-psychology, oncology and CBT. It may also enhance accessibility and improve generalizability across diverse settings, in an international landscape that is evolving in terms of digital health reimbursement strategies (van Kessel et al., 2023).

In some previous trials, control groups were matched based on neuropsychological test scores rather than cognitive complaints or relevant psychopathological variables (Richard et al., 2019). This is an issue because neuropsychology studies (cancer-related or not) (Guerdoux et al., 2009; Paquet et al., 2017; Tucha et al., 2000) frequently report dissociation between the patient-reported neurocognitive problems and the objective performance. By focusing on the perceived cognitive impairment as the primary outcome, this study is in line with growing evidences that subjective complaints are clinically meaningful, often predictive of psychological distress, and may not always correlate with objective deficits. Unlike previous studies that lacked control groups (Maschio et al., 2015; Hassler et al., 2010), our randomized design should allow determining the real impact of the hybrid rehabilitation intervention on the perceived cognitive impairment. This may also help to guarantee sufficient insight and motivation to engage with the intervention. Moreover, the collection of subjective and objective data over time should allow a more nuanced understanding of the relationship between the patients' experiences and their objective neurocognitive performance. In parallel, evaluating adherence, fatigue, psychopathological symptoms, and caregiver-reported outcomes will provide insights into the broader systemic impact of the intervention.

The planned trial has some limitations. First, the absence of a placebo control condition (e.g., same number of telehealth sessions with a non-specialized facilitator and non-therapeutic digital games) limits the ability to identify the specific therapeutic effects of the intervention components. The decision not to include a placebo control group in this trial reflects methodological and ethical considerations. In psychotherapy research, particularly in neuro-oncology, incidental factors (e.g., therapeutic alliance, expectations, and emotional engagement) are not inert, but rather integral to the intervention mechanisms of action (Benedetti et al., 2011; Blanco et al., 2010; Carpenter et al., 2018). Therefore, the design of placebo conditions based on alternative therapies, dismantled interventions, or minimal supportive treatments raises significant concerns. Indeed, this design carries the risk of conflating theoretical models, fails to ensure equivalence in common factors, and may not meet the ethical standards in a medically vulnerable population. In patients with LrGG, in whom cognitive and emotional impairments are clinically relevant and interrelated, a placebo condition would not offer a valid and ethically acceptable comparison. For these reasons, we opted for a real-world control group (usual care). This choice allows an ecologically valid assessment of the added value of the neuro-psychotherapeutic intervention. Non-specific factors, such as attention, perceived support or expectancy effects, may partially explain the observed benefits. The effect size between the intervention and control groups will be discussed in the light of the existing data on placebo effects. For instance, in (Carpenter et al. 2018), the odds ratio of the response rate to CBT compared to placebo was 2.97. Moreover, patients allocated to the control arm will receive standard care according to their center's standard practice. This may include a variety of interventions to reduce cognitive complaints, inducing a variability and thus a risk of bias. However, the existing shortage in Europe of professionals specialized in the cognitive and emotional assessment and management of patients with LrGG before and after surgery (Manso-Ortega et al., 2022) (and possibly even more in the long-term postoperative phase) may contribute to reduce variability within the control group.

Second, the study does not include a measure of pre-morbid stress levels, although baseline stress significantly influences the psycho-oncological needs and coping trajectories in patients with LrGG (Ley et al., 2022). Without this information, interpreting inter-individual variability in outcomes may be more challenging.

Third, the digital neurocognitive tool displays robust psychometric properties in adults with other cancer types (Bray et al., 2017); however, its usability and applicability in patients with LrGG remain to be established. This study will carefully monitor adherence and patient feedback, providing important novel data to address this gap in a real-life cohort.

Fourth, subjective cognitive complaints are influenced by various sociodemographic variables, including age, sex, and socio-cultural level. As these variables will be collected, it will be crucial to include them as covariates in the analyses to take into account their potential confounding effects on the outcomes.

Fifth, the use of psychotropic drugs (e.g., antidepressant, anxiolytic, or antiepileptic drugs) may affect both cognitive performance and emotional wellbeing. As such treatments will not be standardized across participants and may differ between arms despite randomization, they may introduce a bias or variability in the results.

Sixth, all CBT sessions are delivered by the same therapist. This will ensure internal consistency, but may limit the generalizability of results to other clinicians or settings.

Lastly, the hybrid design of this study (CBT + digital tool) will not allow us to disentangle the effects of CBT and of the digital program because they are delivered concomitantly. Also, the hybrid design may lead to the unintentionally exclusion of individuals with limited digital literacy/access. Therefore, the intervention effectiveness could be partly explained by the participants' familiarity with digital technology and the proper functioning of the digital platform. This could limit the generalizability of the findings, especially to more vulnerable or socioeconomically disadvantaged patients.

Despite these limitations, this study represents an important step toward the development of accessible, evidence-based neuropsychological interventions tailored to the specific needs of patients with LrGG. By combining a CBT-informed therapeutic framework with a digital neurocognitive rehabilitation tool, the FREEDOME program offers an innovative, scalable approach to address cognitive complaints and psychological distress in this population. This study targets French-speaking patients. However, most of the used neuropsychological tests, questionnaires, and the digital program have been validated in different languages with standardized guidelines, and the CBT component follows a structured protocol. This will facilitate its international adaptation and implementation. The findings of this trial might inform the future standards of care and might contribute to the broader integration of psycho-oncological support into neuro-oncology care pathways. It may also help to establish models for scalable delivery of psychosocial care in other populations with chronic neurological or oncological conditions.

## 4 Ethics and dissemination

The study protocol (version no. 1.1) was approved by a French national ethics committee, the *Comité de Protection des Personnes Île-de-France XI*, on April 22, 2024. Additionally, the French National Agency for the Safety of Medicines and Health Products (ANSM) was notified on April 25, 2024. Before enrollment, investigators will provide all participants with detailed information about the study objectives and procedures, and written informed consent will be obtained prior to their inclusion in the study. The research will be conducted in accordance with the French Public Health Code (including the Jardé Law), the Declaration of Helsinki, and Good Clinical Practice guidelines. Participants will have the right to access, correct, delete, or restrict the processing of their personal data in compliance with the European Union General Data Protection Regulation. Formal approval from the ethics committee will be required for any substantial amendment to the study protocol. The updated protocol will then be shared with all investigators once approved.

Upon completion of the study, participants may request access to the results. The findings will be disseminated through peer-reviewed journal publications and presentations at national and international conferences.
