# Supplementary material for: Efficacy of hybrid remote neuropsychological rehabilitation on cognitive complaints in post-therapeutic lower-grade glioma: the FREEDOME randomized study protocol
Source: Front Psychol. 2025 Sep 23;16:1650861. doi: 10.3389/fpsyg.2025.1650861 (PMC12500459; doi:10.3389/fpsyg.2025.1650861)
Supplement: Supplementary file 1 [file Data_Sheet_1.pdf]

Formulaire de consentement

Efficacité d'un programme mixte de réhabilitation neuropsychologique en distanciel chez des patients porteurs d'un gliome diffus de grade 2 ou 3 : essai randomisé contrôlé.

FREEDOME

Version n°1.1 du 08/04/2024

N° ICM : PROICM 2023-06 FRE

N° ID-RCB : 2023-A02376-39

Promoteur: **Institut régional du Cancer de Montpellier (ICM)**
208 rue des Apothicaires
34298 Montpellier Cedex 5

Coordonnateur de l'étude: Dr Amélie DARLIX
Co-Coordinateur de l'étude : Dr Estelle GUERDOUX

Je soussigné(e) :

Nom:……………………………. **Prénom**:…………………………………………

Date de naissance: I__I__I I__I__I I__I__I__I__I

certifie avoir lu et compris la note d'information version n° 1.1 du 08/04/2024 qui m'a été remise et accepte de participer à cette recherche selon les conditions définies dans celle-ci.

J'ai bien compris que ma participation à la recherche était libre, et que je pouvais refuser d'y contribuer sans avoir à me justifier, tout en continuant à recevoir la meilleure prise en charge disponible.

Je reconnais avoir pu poser toutes les questions souhaitées et avoir reçu des réponses satisfaisantes à mes questions.

Je reconnais en particulier que le droit à me faire assister par une personne de mon choix m'a été communiqué.

Je reconnais avoir disposé d'un temps de réflexion suffisant entre ces informations et le présent consentement et si je le souhaitais avoir eu l'opportunité d'en discuter avec mon médecin ou mes proches.

Les conditions de ma participation, notamment la durée de celle-ci, les contraintes, les objectifs, le déroulement de l'étude ainsi que les bénéfices et les risques éventuels, m'ont été expliqués clairement par le Dr/Pr…………….

J'ai compris également que je pouvais à tout moment interrompre ma participation à cette recherche, sans avoir à me justifier, sans aucun préjudice et en continuant à recevoir la meilleure prise en charge disponible. Dans ce cas, je m'engage à prévenir le médecin responsable de l'étude.

J'ai eu l'assurance que les décisions qui s'imposent pour ma santé seront prises à tout moment, conformément à l'état actuel des connaissances médicales.

J'ai bien compris que tout fait nouveau susceptible de remettre en cause mon consentement à ma participation à l'étude me serait communiqué.

J'ai bien noté que mon consentement ne dégageait pas les investigateurs et le promoteur de leurs responsabilités, et que je conservais tous les droits qui me sont garantis par la loi.

J'ai bien compris que mes données seront traitées et protégées conformément aux Règlement Européen (RGPD) et à la loi CNIL. J'accepte que les données enregistrées à l'occasion de cette recherche puissent faire l'objet d'un traitement informatisé par le promoteur ou pour son compte, tel que décrit dans la lettre d'information et que les droits concernant mes données personnelles codées s'exercent à tout moment auprès de l'investigateur qui me suit dans le cadre de la recherche et qui connaît mon identité ou du Responsable de la protection des données du promoteur (DPO).

J'ai bien noté, que si je souhaite me retirer de l'étude, les données recueillies avant mon retrait ne pourront pas être supprimées. Par contre, aucune nouvelle donnée ne sera recueillie.
Ces droits s'exercent auprès du médecin qui me suit dans le cadre de cette recherche et qui connaît mon identité.

J'ai pris connaissance que cette recherche a reçu l'accord du Comité de Protection des Personnes Ile de France XI et l'information de l'ANSM.

Je reconnais avoir été informé(e) que le promoteur de l'étude, l'Institut régional du Cancer Montpellier a souscrit une assurance de responsabilité civile pour ce protocole en cas de préjudice auprès de la société RELYENS (contrat n° 140474).

J'ai bien noté que j'ai le droit d'être informé(e) des résultats globaux de cette recherche selon les modalités qui ont été précisées dans le document d'information.

J'atteste être affilié(e) ou bénéficiaire d'un régime français d'assurance maladie (sécurité sociale), condition obligatoire pour pouvoir être inclus dans la recherche.

J'accepte que mes données puissent être réutilisées pour d'autres fins de recherche en neuro-psycho-oncologie	Oui □	Non □
Je consens à ce que mon aidant familial intervienne dans la recherche (via 2 questionnaires)	Oui □	Non □

Nom, Prénom :

Fait à………………. Le └─┴─┘ └─┴─┘ └─┴─┴─┴─┘

Signature du patient :

Nom, Prénom :

Fait à………………. Le └─┴─┘ └─┴─┘ └─┴─┴─┴─┘

Signature de l'investigateur :

Je reconnais qu'un des deux exemplaires de ce formulaire attestant mon consentement m'a été remis.
